# Living with a hidden disability – a qualitative study of post-COVID-19 olfactory dysfunction

**DOI:** 10.3389/falgy.2026.1792460

**Published:** 2026-03-24

**Authors:** Ditte Gertz Mogensen, Pia Dreyer, Vibeke Backer, Mary Jarden

**Affiliations:** 1 Department of Otorhinolaryngology, Head and Neck Surgery & Audiology, Copenhagen University Hospital, Rigshospitalet, Copenhagen, Denmark; 2 Department of Intensive Care, Aarhus University Hospital, Aarhus, Denmark; 3 Institute of Public Health, Section of Nursing, Aarhus University, Aarhus, Denmark; 4 Department of Clinical Medicine, Faculty of Health and Medical Sciences, University of Copenhagen, Copenhagen, Denmark; 5 Department of Hematology, Center for Cancer and Organ Diseases, Copenhagen University Hospital, Rigshospitalet, Copenhagen, Denmark

**Keywords:** COVID-19, lived-experience, long COVID, olfactory dysfunction, quality of life

## Abstract

**Introduction:**

Since the onset of the COVID-19 pandemic, olfactory dysfunction has become increasingly prevalent, manifesting as complete loss of smell, reduced olfactory sensitivity, or distorted perceptions such as parosmia. This qualitative study explored how post-COVID-19 olfactory dysfunction is experienced and how it impacts daily life, well-being, and quality of life.

**Methods:**

A qualitative design inspired by Ricoeur's phenomenological-hermeneutic approach was used. Twenty patients with post-COVID-19 olfactory dysfunction were recruited from an otolaryngology department in Denmark and interviewed between June 2023 and February 2024. Transcribed data were analyzed using Dreyer and Pedersen's Ricoeur-inspired method. The Consolidated Criteria for Reporting Qualitative Research (COREQ) checklist was applied.

**Results:**

The analysis revealed six themes: 1) Smell loss means relying on others’ sense of smell, 2) A sudden fear of body odor, 3) The smell of decay and dreadful taste take over, 4) Losing the pleasure of food and finding comfort in overeating, 5) The loss of the ability to recall and experience meaningful scents, and 6) Living with a hidden disability.

**Conclusions:**

Olfactory dysfunction after COVID-19 is experienced as an invisible yet intrusive condition that impacts daily functioning, eating habits, social relationships and personal identity. The findings highlight the need for greater awareness among healthcare professionals to support patients in developing coping strategies that promote quality of life in the context of long-term sensory loss.

## 1 Introduction

Olfactory dysfunction, defined as a reduced or distorted smell perception, is an underrecognized condition with significant consequences for quality of life and mental health (1, 2). It is classified as either quantitative dysfunction, referring to reduced odor sensitivity (hyposmia) or complete loss of smell (anosmia), or qualitative dysfunction, which involves distortions in odor perception, such as parosmia, where odors are perceived as negatively altered (2, 3). Olfactory dysfunction can be caused by upper respiratory viral infections, sinonasal diseases, facial traumas, neurological disorders, aging, toxic exposures, medications, as well as idiopathic or congenital factors (2). Its prevalence in the general population is estimated to be up to 23%, with higher rates observed among older individuals (4).

Several quantitative studies have shown that olfactory dysfunction is associated with reduced quality of life (1, 5–8). Affected individuals report challenges related to food intake, personal hygiene, safety (e.g., detecting fire or gas leaks), and social and sexual functioning, all of which significantly impact overall well-being (1, 5, 6, 8, 9). However, the literature is not consistent regarding which type of olfactory dysfunction has the greatest impact on quality of life. Some studies suggest that parosmia has the most negative effect on daily life (8, 10, 11), while others highlight that patients with quantitative olfactory dysfunction report the lowest quality of life (12).

Olfactory perception is fundamental to the experience of flavor complexity during meals, as gustatory perception alone primarily detects the basic taste qualities (13). Consequently, both quantitative and qualitative olfactory dysfunction may substantially diminish the sensory enjoyment of food, since meals are often perceived as tasteless or repulsive under such conditions. Such changes may influence appetite regulation and eating behavior (14).

The COVID-19 pandemic has brought increased attention to olfactory dysfunction, as loss of smell was among the most common symptoms during acute SARS-CoV-2 infection, with reported prevalence rates of up to 43% (15). Recent studies estimate that between 2.9% and 8.3% of individuals continue to experience olfactory dysfunction two years post infection (16–18), highlighting its persistence as a common post-COVID-19 condition. Interestingly, post-COVID-19 olfactory dysfunction appears to differ from other post-infectious cases, as it is more common among middled-aged individuals, women, and those with mild or asymptomatic infections. Moreover, it is associated with a higher prevalence of parosmia (19, 20).

Although previous research demonstrates that olfactory dysfunction reduces quality of life, only a few qualitative studies have investigated how individuals experience living with this condition (21, 22). To date, no qualitative studies have examined the lived experiences of individuals suffering from post-COVID-19 olfactory dysfunction or parosmia, despite evidence that this condition differs from other post-infectious olfactory disorders in terms of age and sex (19, 20). Understanding these experiences is essential for developing patient-centered interventions and improving care strategies for this patient group. This study therefore aimed to explore the experiences of olfactory dysfunction as a post-COVID-19 condition and to examine its impact on daily life, well-being, and quality of life.

## 2 Materials and methods

### 2.1 Design

To explore the experiences of post-COVID-19 olfactory dysfunction, a qualitative study was conducted using a phenomenological-hermeneutic approach (23), inspired by Paul Ricoeur's theory of interpretation (24). This approach was selected for its ability to capture phenomena as they are lived and to provide an in-depth understanding of human experience (24, 25). To ensure methodological rigor and reflexivity, the study adhered to the Consolidated Criteria for Reporting Qualitative Research (COREQ) checklist (26).

### 2.2 Setting and participants

Participants were patients referred to an otolaryngology department in Denmark due to post-COVID-19 olfactory dysfunction. As a part of their standard clinical evaluation, all participants underwent an assessment in accordance with the position paper on olfactory dysfunction (2). This included a detailed medical history, rhinoscopy, and psychophysical olfactory function testing using the Burghart “Sniffin’ Sticks” extended test with *n*-butanol. This test objectively measures the odor threshold (T), odor discrimination (D), and odor identification (I), generating a total TDI score (27). In addition, patients were routinely informed about available treatment options for olfactory dysfunction during this standard assessment. Inclusion criteria were age 18 years or older, diagnosis of post-COVID-19 olfactory dysfunction by an otolaryngologist, and a TDI score ≤30.75 and/or a diagnosis of parosmia based on medical history. Exclusion criteria included inability to understand and speak Danish and olfactory dysfunction attributed to conditions other than COVID-19. If patients met the inclusion criteria, the doctor and nurse who examined them provided written participant information. Those who expressed interest were contacted by the first author, either immediately, after the examination or by phone. Participants were then selected through purposeful sampling, with focus on variation in age, gender, and type of olfactory dysfunction to ensure diverse perspectives and nuanced descriptions of the phenomenon (28, 29). The study included 20 participants (15 women and 5 men) aged 22–78 years (Table 1).

**Table 1:** Characteristics of participants.

Participants	Gender	Age	Type of olfactory dysfunction	Time after infection (months)	Type of interview
1	Female	22	Hyposmia	27	In-person
2	Female	32	Hyposmia+parosmia	39	In-person
3	Female	33	Hyposmia+parosmia	26	Telephone
4	Female	41	Hyposmia+parosmia	38	In-person
5	Female	43	Hyposmia	25	Telephone
6	Female	47	Anosmia	28	Telephone
7	Female	48	Hyposmia+parosmia	23	Telephone
8	Female	54	Hyposmia+parosmia	14	In-person
9	Female	58	Anosmia	29	In-person
10	Female	59	Hyposmia+parosmia	30	In-person
11	Female	60	Hyposmia+parosmia	23	Telephone
12	Female	60	Hyposmia	35	In-person
13	Female	62	Hyposmia+parosmia	33	Telephone
14	Female	63	Hyposmia+parosmia	12	In-person
15	Female	63	Hyposmia	25	Telephone
16	Male	22	Anosmia	36	Telephone
17	Male	35	Parosmia	20	In-person
18	Male	48	Hyposmia+parosmia	35	Telephone
19	Male	73	Hyposmia	29	Telephone
20	Male	78	Anosmia	24	In-person

Time since infection refers to the COVID-19 infection associated with symptom onset.

### 2.3 Data collection

Data were collected through qualitative semi-structured interviews, inspired by Brinkmann & Kvale (28). The interviews were conducted individually by the first author between June 2023 and February 2024. The interview guide, developed by the first and last authors based on relevant literature and theory, was pilot tested with one participant, resulting in minor adjustments. Interviews began with the question “Can you tell me about the time you found out you had Corona?” to encourage participants to share their initial experiences with COVID-19 symptoms. Subsequent questions addressed their experiences with olfactory dysfunction as a post-COVID-19 condition, including “How has your impaired sense of smell affected your everyday life?” and “How did you manage having an impaired sense of smell?”. Probing questions such as “Can you elaborate on that?” were used to elicit more detailed and nuanced narratives. The interviewer aimed to create a supportive environment where participants could freely express their experiences without interruption (28). In total, 20 interviews were conducted, each lasting between 20 and 50 min. Participants were able to choose their preferred date, time, and interview format to accommodate their schedules and personal comfort. Ten interviews were conducted in person in a quiet, undisturbed room at the department, and 10 were conducted by telephone (Table 1). All interviews were audio-recorded and transcribed verbatim.

### 2.4 Data analysis

The analysis followed Dreyer & Pedersen's phenomenological hermeneutical method, inspired by Paul Ricoeur, which involved a dialectical process between understanding and explanation, consisting of three steps: (i) naïve reading; (ii) structural analysis; and (iii) critical analysis and discussion (23). The first step, naïve reading, involved an initial reading of all interview texts to gain a general sense of the overall text. The second step, structural analysis, consisted of three levels of interpretation, moving between the parts and the whole in a hermeneutic circle (Table 2). The first level consisted of an interpretation of the entire dataset, the 20 interview texts, by identifying meaning-bearing units (what is said). The second level involved a dialectic movement from understanding “what the text said” to a deeper understanding of “what the text speaks about”, focusing on significance-bearing units (23). The third level involved identification and formulation of themes based on the meaning-bearing and significance-bearing units from the previous levels. The final step was the critical analysis and discussion of the identified themes, involving a dialectical process between explanation and interpretation (23). During this step, findings were discussed in the context of relevant literature and research to deepen the understanding of the phenomenon.

**Table 2:** An example of the structural analysis.

Meaning-bearing units (What is said)	Significance-bearing units (What the text talks about)	Theme
*"Well, it's a hidden disability that's different than if you can't hear, you can get a hearing aid, then you can hear a little bit again, right? Or there are glasses. I mean, there aren't really any adjustments that can be made”* (P18) *“I have also sometimes doubted whether people believed it, because I am the only one who can feel it and sense it, taste it and smell it. So, it must be difficult for others to relate to it. And maybe others also think it's just imaginary”* (P17) *“I have not been heard by my doctor or accepted. I haven't gotten any support. My doctor just says that I can have a “taste party” when I can taste again. I feel I've been very alone in this”* (P1)	Losing the sense of smell is experienced as a hidden disability, one that is invisible to others. It is described that others cannot see that “there is something wrong with me,” and it therefore feels overlooked and misunderstood by those around. People around them often struggle to grasp the impact of losing the sense of smell, and its importance is only realized once it is gone. The disability is faced alone, with limited ways to address it or receive help. As a sensory loss, there are no real adjustments that can be made. The absence of visible signs and concrete aids intensifies feelings of doubt, for both the person and those around them. When only the person affected can feel the sensory loss, and it remains invisible to others, doubt may arise about whether others understand and believe their experience …	Living with a hidden disability

The analysis was conducted by the first author using the software program NVivo (v. 14, QRS International Pty Ltd., Victoria, Australia). The dataset comprised 20 interview transcripts, totalling 129 pages. The analysis was reviewed and verified by all authors.

### 2.5 Ethical considerations

In accordance with the Helsinki Declaration (30), all participants received oral and written information about the interview study before providing written informed consent. They were informed that participation was voluntary, that they could withdraw at any time without explanation, and that declining to participate would have no consequences. The study was approved by Scientific Ethics Committees for the Capital Region of Denmark (H-22038919) and by the Capital Region of Denmark's research documentation, Pactius (P-2022-476).

## 3 Findings

### 3.1 Naïve Reading

Participants experienced post-COVID-19 olfactory dysfunction as a disabling condition that significantly reduces quality of life. The loss of this sense negatively affects mood, family life and social relationships. Enjoyment in preparing and eating food has diminished or, for some, disappeared completely, and social interactions involving food and dining out have been altered. Changes in the perception of body odors complicate assessing personal hygiene and recognizing loved ones by smell. Participants describe their experience as living with a disability where the joy of life has disappeared. They must suddenly rely on others’ sense of smell to assess body odor, food freshness and detect danger signals, which requires adaptation to a new way of living after previously being self-reliant.

### 3.2 Structural analysis

Through the structural analysis, six themes emerged.

#### 3.2.1 Smell loss means relying on others’ sense of smell

Participants experienced that when the sense of smell disappears, they become dependent on others to ‘smell’ for them. This is described as having a close friend, family member or colleague become their nose or borrowing someone else's sense of smell. Such dependence can feel overwhelming, especially for those used to being independent. Relying on others to detect environmental odors become necessary when self-monitoring is no longer possible. One participant describes this experience as follows:

“Some of my independence has been taken away. Not being able to smell if something is too bad – I've had to borrow others’ sense of smell. You suddenly become dependent on other people” (P3)

It can be very uncomfortable and worrying when the sense of smell fails to detect danger signals such as fire, smoke or gas. Suddenly, one becomes dependent on others to detect these danger signals, as it is no longer possible to recognize them independently. In hazardous situations, other senses are heightened, particularly sight, where dangers are only noticed when visible to the naked eye. This is perceived as if the brain is not functioning properly and can no longer smell danger, which is especially worrying if a threat arises suddenly:

“I can't smell smoke. At one point, there was a fire in our kitchen at work, something had caught fire, and I couldn't smell it until I saw it. It's just a big concern subconsciously that you can't smell warning signs” (P2)

Uncertainty arises when judging the freshness of food, as participants can no longer detect if it smells wrong. Participants describe realizing food was spoiled only after others noticed its bad taste. In these situations, they lose trust in their senses, becoming more cautious and discard food when in doubt. One participant describes it as follows:

“I've had some experiences where I've eaten something and the people, I've eaten with have realized that it tastes really bad, spoiled. And I haven't sensed that. So, I can see that I'm staying away from shrimp and things like that” (P4)

Both personal and professional life are affected when the sense of smell disappears. It is frustrating and problematic when the sense of smell no longer works, especially when it is relied upon in a professional setting. Participants find it emotionally stressful to explain and defend their condition fearing they may be seen as unprofessional:

“When I'm at work in the daycare, I've experienced parents coming to pick up their child who's sitting with me wearing a dirty diaper. They get really upset that I haven't done anything. But I just don't notice it myself” (P5)

In their professional life, they have suddenly become dependent on colleagues to identify alcohol abuse, dirty diapers and other situations that require a well-functioning sense of smell.

#### 3.2.2 A sudden fear of body odor

When the sense of smell disappears, it creates uncertainty about one's own and others’ body odors and personal hygiene. Participants describe no longer knowing if they themselves have an odor, making it necessary to rely on others or develop strategies to avoid embarrassing or unhygienic situations. This leads to increased vigilance in personal grooming because there is no longer a sense of one's own body odor. Being in public without knowing if one's clothes smell is described as both irritating and worrying. One participant expresses it at follows:

“You don't really know when you smell yourself. I've asked my friends to tell me if I smell. Even if you shower every day, you might smell, and you just can't tell. It's also annoying that you don't really know if you're wearing a t-shirt that smells” (P16)

Although participants shower daily and previously had no problems with smelling of sweat, a sudden concern arises because they can no longer smell it themselves and worry whether others will tell them. They can no longer smell that they do not smell, so they seek reassurance from others. Even when assured they do not smell, doubts can still arise, leading to constant worry about body odor and the feeling of walking around smelling unpleasant. Doubting one's own hygiene and body odor is uncomfortable. The fear of having bad breath without being able to smell it personally leads some to chew gum to avoid embarrassing situations. One participant describes the experience as follows:

“It's very uncomfortable. I always have chewing gum because I think, at least I won't smell. I constantly have to have it with me. I would be really sad if others could smell the taste I have” (P10)

Common actions, such as reaching over others, are avoided for fear of smelling sweat. There is an increased awareness to avoid situations where they come too close to others because they are afraid of smelling. When unable to determine if clothes are clean or smell of sweat, they tend to wash them more often than necessary:

“I am very conscious about smelling of sweat, even though I have never been someone who smelled. If I have to reach over something, I think it is unpleasant for others. I am quite affected by it. I also wash much more clothes than I probably really need to” (P2)

Worrying about their own body odor alter how they interact with others and increases awareness of personal hygiene.

#### 3.2.3 The smell of decay and dreadful tastes take over

The sense of smell has changed, and things no longer taste or smell as they once did. Sensory perceptions are distorted, causing everything to taste and smell strange, described as if everything is decaying. Participants report having to spit out food because it tastes awful and unpleasant, no longer matching their expectations. The smell of brewing coffee can be extremely unpleasant and raise doubts about whether it should be consumed. Foods that were once enjoyed may suddenly smell and taste bad, leading to avoidance and a loss of appetite. One participant describes it as follows:

“I really felt that everything was decaying, really everything. And it's horrible to sit and eat something and it just tastes so bad. Sometimes I just have to spit it out” (P12)

Scents that once smelled pleasant now smell sour and strange, leading them to wonder whether this is their true perception, or a distortion created by the brain. They experience strong and unpleasant odors that are difficult to identify but clearly present. Strong scents, like perfume, can suddenly become intensified, causing nausea and headaches. Being in crowded places where many people wear perfume is described as uncomfortable and can make one feel sick. One participant describes the experience as follows:

“If I walk through a strong smell, there is a kind of nasty smell. The bad smell is the same no matter what strong odor I pass by. I wonder what this smells like. I don't know if its heavy arm sweat or perfume” (P14)

The distorted olfactory experiences, specifically parosmia, are described by participants using the metaphor of visual hallucinations, where people feel that what they see is genuinely real, yet simultaneously recognize it is not. There is a sense that their brain is playing tricks on them. It is described as cola tasting and smelling like burnt plastic, and other foods like rot, even though they know this isn't true. The brain has created an altered reality, making it feel as though they are consuming something toxic. One participant describes this experience figuratively:

I compare it to people with hallucinations who suddenly see a snake crawling down the wall – it feels completely real and vivid for them. You might think they need to pull themselves together, but their brain tells them it's real. My brain has completely messed with me too. It's like eating burnt plastic if I have raw onions or cola. Others say it tastes wonderful, but not for me, not in my head right now at least. The brain can really create a different picture of reality (P18)

For others, it makes no sense, but the participants have had to accept that this is how it is experienced now, that their distorted sense of smell provides a different experience of reality.

#### 3.2.4 Losing the pleasure of food and finding comfort in overeating

The joy of cooking and eating fades when the sense of smell no longer functions as before. Cooking loses its appeal and becomes a meaningless and tedious chore, no longer driven by desire. Eating turns into a monotonous experience, with food tasting of nothing and mealtimes feeling boring. The texture of food becomes extremely important, as it must avoid being mushy and bland. One participant describes this experience as follows:

“I liked to cook and bake. But I don't have that in me anymore, because I can't taste it. Well, half of my life and my desire for food has disappeared. And it affects the other three at home” (P6)

This illustrates how the loss of olfactory function can disrupt familiar household roles, reduce participation in shared activities, and create a sense of limitation or burden within the family.

The seasoning of the food has changed because only the basic tastes of sour, sweet, salty, and bitter can be detected. Meals are often over salted in an attempt to add some flavor. There is also an increased craving for sugar, leading to the exhausting experience of constantly needing to eat unhealthy foods. Consuming sugar becomes a way to stimulate oneself, because one is seeking pleasure in a new way. One participant describes how eating unhealthily has become a reality:

“Gaining weight is exhausting because I eat so much sugar. I get so focused on it because nothing really tastes of anything, so everything is extremely boring. I also salt my food too much, just to give it some flavor” (P9)

Although the desire to eat has disappeared because the food lacks flavor, participants sometimes experience episodes of overeating, driven by the hope that the next bite will tastes like something. This reflects a desperate attempt to regain the sense of flavor and food pleasure they miss. The overeating and increased craving for sugar lead to weight gain, which feels uncomfortable and difficult to control. One participant describes:

“I actually felt for a while that I was eating more. Because I become so desperate to get the oral stimulation that comes with it. And I've had to stop that now” (P7)

Participants have withdrawn from social interactions involving meals and no longer go out to eat or drink coffee with family and friends. This withdrawal leads to feelings of isolation and social disconnection, as they are increasingly excluded from social events involving food after repeatedly declining invitations. One participant describes this experience as follows:

“It quickly started saying thank you for the invitation, but it will be without me. I'm a bit sad when I hear the guys have been to a wine tasting. And of course, it's good that they don't keep calling and asking if I want to come along, because that's rubs salt in the wound” (P18)

Although participants feel a sense of sadness about missing out on social events, they also experience relief in not having to face the reality of what they are missing.

#### 3.2.5 The loss of the ability to recall and experience meaningful scents

When the sense of smell is impaired or lost entirely, an important sensory dimension of life is suddenly missing. This creates a feeling that the familiar world has changed, and that sensory input is missing. One participant describes it as follows:

“It feels like having a grey veil in front of my nose and mouth… I lack input. There is a sensory dimension in my life that is simply missing” (P14)

When a sense disappears, a part of the overall experience is lost. A walk in the woods can still be beautiful, but without the familiar scents, the experience becomes different and the connection to the surroundings weakens. There is a longing for the scent of wet asphalt, rain, lilacs, mom's homemade cooking, and the familiar smells once taken for granted but now missed, affecting quality of life. Places, events and people are remembered through scents, and when the sense of smell is lost, an anxiety of forgetting these memories arises. One participant describes what it feels like to miss these meaningful scents:

“It's really boring, not having the joy of smelling. One thing is smells, but also those lovely scents. That's what's nice about going out when the rain has hit the asphalt. Things like that, which you've always appreciated, or going out to the sea. You almost miss the smell of seaweed. That sense of perception is gone, and that affects me” (P4)

The loss of sense of smell impairs the ability to experience emotional and intimate moments with loved ones. The scent of parents, children, and close family is closely associated with feelings of safety and love, and its absence creates a sense of longing and sorrow. Participants describe striving to find joy in the rare moments when their sense of smell briefly returns. They enjoy the brief moments when a familiar scent suddenly appears, even if it quickly disappears again. One participant expresses this as follows:

“The biggest thing is not being able to hug your parents and smell them, feeling that sense of safety. I try not to think too much about it, because it becomes a black hole. I try to enjoy when I can suddenly smell something, like when I hug my brother, and I can suddenly smell him, but then it's just gone again” (P2)

Another participant describes the experience of suddenly no longer being able to smell their child:

“The first year, I was quite affected. Mainly because I had a three-year-old daughter, and the thought of never being able to smell her again. I was completely devastated by that. Now I've come to terms with it, because I still can't smell my child” (P5)

It is a process of coming to terms with the loss, despite ongoing frustrations over what is missing. Notably, the inability to smell one's children is experienced as a great sorrow.

#### 3.2.6 Living with a hidden disability

Losing the sense of smell is experienced as a hidden disability, one that is invisible to others. It is described that others cannot see that “there is something wrong with me,” and it therefore feels overlooked and misunderstood by those around. People around them often struggle to grasp the impact of losing the sense of smell, and its importance is only realized once it is gone. The disability is faced alone, with limited ways to address it or receive help. As a sensory loss, there are no real adjustments that can be made. A participant describes it as follows:

“Well, it's a hidden disability that's different than if you can't hear, you can get hearing aids, then you can hear a little bit again, right? Or there are glasses. I mean, there aren't really any adjustments that can be made” (P18)

The absence of visible signs and concrete aids intensifies feelings of doubt, for both the person and those around them. When only the person affected can feel the sensory loss, and it remains invisible to others, doubt may arise about whether others understand and believe their experience:

“I have also sometimes doubted whether people believed it, because I am the only one who can feel it, sense it, taste it and smell it. So, it must be difficult for others to relate to it. And maybe others also think it's just imaginary” (P17)

When the loss of the sense of smell is not visible to others, there is a feeling of not being taken seriously. There is a fear that others may perceive the condition as imaginary, unlike visible illnesses such as a broken leg. Participants describe that not only those close to them but also healthcare professionals often struggle to understand or acknowledge the sensory loss. One participant describes how their doctor did not take the condition seriously:

“I have not been heard by my doctor or accepted. I haven't gotten any support. My doctor just says that I can have a “taste party” when I can taste again. I feel I've been very alone in this” (P1)

## 4 Discussion

The findings of this study provide a nuanced understanding of olfactory dysfunction as a complex post-COVID-19 condition, manifesting not only as a physical sensory loss but also as a condition with significant psychosocial consequences. This section draws on relevant theoretical perspectives and existing research to interpret the findings of the study, aiming to achieve an in-depth understanding of the phenomenon.

A key challenge for individuals with post-COVID-19 olfactory dysfunction is that the condition is experienced as a hidden disability often misunderstood or dismissed by others, including healthcare professionals. This aligns with previous studies on non-COVID-19 olfactory dysfunction, where participants reported that their condition was often neglected or met with disinterest, particularly by healthcare professionals (21, 22), suggesting that this issue extends beyond the COVID-19 context. Research on other invisible conditions such as fibromyalgia and chronic fatigue syndrome, similarly shows that patients often feel disbelieved due to the absence of visible symptoms (31, 32). According to Goffman's theory of stigma, individuals with olfactory dysfunction may experience *discrediting stigma,* as the invisibility of the condition places them in a vulnerable position where their condition is not readily recognized as legitimate (33). Consequently, they may feel pressured to justify their condition in social, professional, and clinical contexts, as reflected in the findings. This ongoing need for explanation can be emotionally exhausting, leading some to conceal their condition to avoid doubt or social judgment (33). This highlights the need for greater awareness and sensitivity in clinical practice, particularly regarding conditions characterized by invisible symptoms.

Another central finding was a loss of confidence in the sense of smell, which reduced perceived safety and led to behavioral changes. These included heightened awareness of potential dangers such as fire or spoiled food, frequent hygiene routines to avoid body odor, avoidance of social dining, and reliance on others to confirm sensory impressions. These behaviors can be understood through Lazarus and Folkman's (34) coping theory, which defines coping as the cognitive and behavioral efforts individuals use to manage stressful demands. Practical adjustments such as increased attention to hygiene, food safety and fire safety, as well as seeking reassurance from others reflect *problem-focused coping*, aimed at managing specific, practical challenges. In contrast, behaviors such as withdrawing from social events to avoid confronting their loss of smell or overeating, particularly sweets to achieve some kind of stimuli, illustrate *emotion-focused coping*, where individuals attempt to regulate emotional distress (34). While these strategies of social isolation and binge eating may provide short-term relief, especially in emotionally taxing situations, they risk contributing to long-term physical and mental health problems.

This study demonstrated that impaired olfactory function causes practical, social, and emotional challenges, consistent with previous quantitative research showing that olfactory dysfunction negatively impacts quality of life. Studies indicate that olfactory dysfunction can impair food intake, reduce the enjoyment of eating, affect personal hygiene and safety, disrupt social relationships, and is associated with an increased risk of depression (1, 5, 8, 9, 11). Similar concerns were reported by participants in a qualitative study based on written patient accounts, which found that reduced olfactory function exposes individuals to potential hazards and negatively impacts a wide range of everyday activities (21). These findings support the view that olfactory dysfunction constitutes a condition with substantial implications for daily functioning and mental health.

Unique for this qualitative study was the inclusion of patients with parosmia, who perceived olfactory distortions as a symptom that caused doubt about whether the issue was with the nose or the brain. This led to feelings of confusion and insecurity. Some even likened the experience to hallucinations, unsure whether what they sensed was real. These experiences can be understood in relation to the concept of *sensory deprivation*, which suggests that the absence or disruption of sensory input can lead to disorientation, altered self-perception, and emotional distress (35). When the body no longer senses the environment as before, and one cannot trust whether the food is spoiled, smoke is present, or if they smell of sweat or perfume, this uncertainty may, according to Zubek (35), lead to over-controlling behavior, such as excessive showering and washing. Over time, such behaviors can cause psychological strain and negatively affect overall well-being. Olfactory dysfunction should therefore be understood not merely as a sensory loss, but as a condition that can alter one's relationship to the body and the surrounding world. The comparison to hallucinations suggests a blurred boundary between perception and reality, which may contribute to experiences of alienation, uncertainty, and loss of control.

Becoming dependent on others after losing the sense of smell emerged as another central finding. To “borrow others’ noses” to determine whether a t-shirt smelled of sweat, if a diaper needed changing, or if the food they were about to eat was fresh suddenly became necessary. This experience highlights how olfactory dysfunction can compromise an individual's sense of autonomy and self-reliance, similar to the impact of other sensory impairments on independence (36–38).

Previous research indicates that sensory loss can alter self-perception and evoke grief over the loss of sensory experiences (22, 36). Similar findings were observed in the present study, where the loss of the sense of smell resulted in a profound longing that extended beyond its physical function. The inability to perceive the scent of one's children or close family members was described as a source of sorrow, underscoring how olfactory dysfunction disrupts emotional intimacy and relational connectedness. Moreover, the fear of forgetting meaningful scents further illustrates the deep connection between smell, memory, and identity. These findings suggest that olfactory dysfunction should not be understood solely as a physiological impairment but also as a condition with existential and emotional dimensions.

### 4.1 Strengths and limitations

A limitation of this study is that most participants had lived with olfactory dysfunction for over two years since their COVID-19 infection. Including individuals with more recent onset might have provided a different understanding, as their experiences could differ with only a short experience of living with the phenomenon. However, the primary aim of this study was to explore participants’ experiences of living with olfactory dysfunction, and despite the long symptom history, participants were able to reflect meaningfully on their experiences, which remained the central focus of the study. Although purposeful sampling was used, with a focus on variation in age, gender, and type of olfactory dysfunction, the sample still showed relative homogeneity, being predominantly women aged 40 to 65. This reflects the characteristics of the patient population with post-COVID-19 olfactory dysfunction, as previous studies have shown that middle-aged women are most likely to report this condition (9, 17, 19). Nevertheless, this sample may be too small, as the participants included in the study are too homogeneous and may not fully grasp variation in lived-experiences of all individuals with post-COVID-19 olfactory dysfunction.

The use of mixed interview modalities, both in-person and telephone, could theoretically introduce differences in level of detail, emotional expression, and narrative depth. In our study, however, we did not observe any meaningful differences in the depth or quality of interviews between the two formats. It is even possible that participants who chose telephone interviews found it easier to express themselves openly, highlighting the value of allowing participants to select their preferred interview format.

A major strength of the study is that it sheds light on a previously unexplored area, as no prior interview-based research has investigated the lived experience of post-COVID-19 olfactory dysfunction, notably incorporating the perspective of patients with parosmia. All participants described their participation in the study as meaningful, and for some, the opportunity to share their experiences as therapeutic. Another strength was the open and narrative approach to the interviews, which ensured an open phenomenological approach to data collection (28). In addition, the trustworthiness of the analysis was enhanced through independent coding, regular consensus discussions among the author group, and maintenance of an audit trail (as presented in Table 2). Finally, one of the authors (PD) developed the Ricoeur-inspired method used in the analysis. Her expertise supported a deeper level of analysis and contributed substantially to the interpretation of the data.

### 4.2 Implications for practice

The findings demonstrate how loss of smell can significantly disrupt daily life and negatively impact overall well-being. Healthcare professionals should be aware that patients with olfactory dysfunction may experience distress and reduced quality of life, and it is therefore essential to actively inquire about and acknowledge these often invisible symptoms. Patient's experiences must be validated, and effective management strategies should be provided to address both symptoms and concerns. At the same time, individuals should be supported in developing sustainable coping mechanisms that promote a healthier lifestyle, enhance overall well-being, and active social participation. These strategies can include dietary guidance to ensure proper nutrition despite taste distortions, hygiene counseling to alleviate concerns or excessive personal hygiene, and psychological support to help prevent depression and social isolation. One potential approach is the development of a patient education programs, offering interdisciplinary guidance and opportunities to meet others facing similar challenges.

## 5 Conclusion

The study provided an in-depth insight into living with post-COVID-19 olfactory dysfunction. Loss and distortion of smell are experienced as an invisible yet intrusive condition, that not only affects practical aspects of everyday life, but also leads to unhealthy eating habits, affects independence, trust in one's own body and social relationships. The fear of losing meaningful scents, such as those of loved ones, shows how closely the sense of smell is connected to memory, identity and intimacy. The findings thus contribute to a nuanced understanding of olfactory dysfunction as a complex, embodied and relational phenomenon. The study highlights the need for increased awareness among healthcare professionals to support patients in developing coping strategies that promote a healthy lifestyle, well-being and quality of life despite long-term sensory loss.

## Data Availability

The datasets presented in this article are not readily available because the participants of this study did not give written consent for their data to be shared publicly, so due to the sensitive nature of the research supporting data is not available. Requests to access the datasets should be directed to Ditte Mogensen, ditte.gertz.mogensen@regionh.dk.
